# Traplines: A Promising Modification to Reduce Sea Turtle Bycatch in Swordfish Longlines

**DOI:** 10.3390/ani16152295

**Published:** 2026-07-24

**Authors:** Paolo Casale, Vittoria Fiandino, Daniela Freggi

**Affiliations:** 1 Department of Biology, University of Pisa, 56126 Pisa, Italy; vittoria.fiandino@phd.unipi.it; 2 Associazione Caretta Caretta, 92031 Lampedusa and Linosa, Italy; freggidaniela@gmail.com

**Keywords:** loggerhead turtle, *Caretta caretta*, fisheries mitigation, Mediterranean Sea, bycatch reduction, gear modification

## Abstract

Fisheries bycatch is a major threat to sea turtles, and drifting longlines are among the most impactful gears worldwide; however, existing mitigation measures vary in effectiveness and may involve trade-offs with commercial catch volumes. Traplines are a new hookless terminal gear recently adopted in some pelagic longline fisheries, but their conservation value for sea turtles has not been assessed yet. We compared traplines and conventional J-hooks in 23 pelagic longline sets conducted by a single vessel in the central Mediterranean targeting swordfish. Within each set, hooks and traplines were deployed alternately along the same drifting longline, allowing paired comparison under identical conditions. Across all sets, 40,800 hooks and 6850 traplines were deployed. Hooks caught 127 loggerhead turtles (*Caretta caretta*) and 9711 kg of commercial fish, whereas traplines caught 1 turtle and 6580 kg of fish. Turtle and fish catch rates were significantly lower and higher for traplines, respectively. Conservation efficiency relative to economic performance was assessed as turtles caught per kilogram of fish, and this ratio was significantly lower for traplines. These preliminary results provide the first empirical evidence that traplines can nearly eliminate sea turtle bycatch while still producing substantial fish catches, at least under the environmental and technical conditions of this study. We encourage further research in a variety of contexts to assess the broader value of traplines for sea turtle conservation.

## Simple Summary

Sea turtles are often accidentally caught in longline fisheries, where many baited hooks are set in the open sea to catch large fish such as swordfish. This is a serious conservation problem because captured turtles can be injured or die. Current methods to reduce this problem do not always work well and can sometimes reduce fish catches. In this study, we tested a new hookless fishing device called a trapline, which is designed to catch fish without using a hook. We compared traplines with traditional hooks during 23 fishing trips in the central Mediterranean Sea. The traditional hooks caught 127 loggerhead turtles, while traplines caught only 1 turtle. At the same time, traplines caught a larger amount of commercial fish for each fishing device deployed. This means that traplines greatly reduced turtle bycatch while still allowing fishing to continue. These are encouraging early results and suggest that traplines could become an important new tool for protecting sea turtles. More studies are needed in other areas and fishing conditions to confirm how broadly effective traplines are, but this approach could help make fisheries more sustainable and reduce harm to threatened marine wildlife.

## 1. Introduction

Fisheries bycatch represents a major anthropogenic threat to many large marine vertebrates of conservation concern [1,2], including sea turtles [3,4]. Drifting longlines are among the most impactful fishing gears globally and represent a serious conservation issue for sea turtles [5,6,7]. Drifting longlines primarily target highly migratory pelagic species such as tunas, billfishes, sharks and dolphinfishes [8,9]. However, drifting longlines are also responsible for substantial levels of bycatch [1], including sea turtle [10,11,12].

Except in some long-duration deep-set longline operations [13], where substantial mortality may occur before retrieval, sea turtle mortality mainly occurs after release due to ingestion of the hook and, in particular, the attached branchline, which can impair intestinal peristalsis [14,15]. This post-release mortality remains poorly quantified because it is difficult to assess under field conditions. However, available evidence suggests that it may exceed 30% in the worst case [14,16].

Several mitigation measures have been proposed to reduce the impact of drifting longlines on sea turtle populations. These can be broadly grouped into three approaches: (i) reducing the probability of interaction, (ii) reducing the probability of hook ingestion/capture, and (iii) reducing post-release mortality. The first approach involves setting hooks at depths not typically frequented by turtles. Significant reductions in turtle bycatch rate (up to 99.5%) have been reported, but this approach may increase interactions with other sensitive species [17,18,19]. The second approach aims to reduce hook ingestion—and the probability of capture—by using larger hooks and baits that are more difficult for turtles to swallow. For example, replacing squid bait with larger fish bait (i.e., large mackerel) has been shown to reduce hook ingestion and sea turtle bycatch, with reported reductions ranging from 43% to 72% [7,20,21,22]. However, effectiveness varies among turtle species [6], and may entail trade-offs, including reduced catch of some target species [23,24] or increased bycatch of other vulnerable taxa [25,26]. Similarly, replacing traditional J-hooks with larger circle hooks has been proposed to reduce turtle bycatch, with reported reductions of 14–61% [21]. However, their effectiveness in reducing turtle bycatch rate varies across fishing grounds and sea turtle species [27,28] and in some fleets, circle hooks have been associated with lower target species catch [27,29]. As a third approach, circle hooks (irrespective of size) have also been proposed as a means to reduce post-release mortality, as they are more likely to lodge in the mouth and therefore be removed by fishers [29,30,31]. However, this assumes that fishers take the time to remove the hook, despite circle hooks often being more difficult to disengage than J-hooks [32]. If turtles are instead released with the hook and part of the branchline still attached, the expected reduction in post-release mortality may not occur, given the harmful effects of the retained branchline. Overall, the performance of existing mitigation measures is strongly context-dependent, and none can be considered universally effective across fleets, regions, target species, and sea turtle species.

The trapline is a recently introduced terminal gear alternative to hooks that is used in drifting longline fisheries targeting swordfish (*Xiphias gladius* Linnaeus, 1758) [33]. It derives from the Meka-ring (or “ring-shaped branchline”), a hookless gear developed in Japan in the early 2000s and originally inspired by fishing techniques used in the diamondback squid (*Thysanoteuthis rhombus* Troschel, 1857) fishery [34,35]. Since at least 2021, this gear has been used in Spanish fleets and is currently adopted by several fleets operating in both the Mediterranean Sea and the Atlantic Ocean [36,37,38,39]. Recently, ICCAT adopted a recommendation (Rec. 25-09) allowing the use of traplines, provided that standardized data are collected to assess their performance.

In its most common configuration, the trapline consists of multiple concentric loops—typically seven, but ranging from four to eight—of variable size (140–210 cm in circumference), made of transparent nylon or polyamide monofilament. The bait, if used, is positioned centrally within the loop system [37,38]. The loops are held together at the upper end by crimp sleeves and connected to the main line through secondary branchlines analogous to those used for hooks. This configuration allows traplines to be deployed alternately with conventional hooks along the longline, at variable ratios depending on fishers’ operational preferences [36,37,38]. Artificial bait, most commonly resembling mackerel or squid, is generally used. Additional elements such as light sources (LED or chemical lightstick) or organic bait may be included at the fisher’s discretion, although these components do not appear to be essential for the functioning of the gear [33,37,38]. A trapline is therefore a hookless terminal capture device rather than a passive attractor. Its capture mechanism differs from that of a conventional hook: instead of capturing animals through ingestion or external hooking, the target fish is retained when it passes into the loop system and one or more loops become caught around body structures. Ochi et al. [35] described this mechanism for the Japanese meka-ring, reporting that swordfish were usually caught with the head and pectoral region inside the loops, with loops retained around the rostrum, jaws, and dorsal or pectoral fins. This capture process is thought to be facilitated by swordfish morphology, because swordfish cannot fold the first dorsal and pectoral fins flat against the body, making escape difficult once the fish is retained in the loops [35]. Each trapline functions as a single terminal capture unit and can retain at most one fish or other animal at a time.

Preliminary technical reports, mostly from recent ICCAT working documents, suggest that traplines may produce different catch outcomes compared to traditional drifting longlines. Reported differences include potential increases in catch rates and mean size of target species, together with reduced bycatch of several vulnerable taxa [36,37,38]. Because traplines are a recently adopted gear, peer-reviewed evidence remains limited, and these reports should be interpreted as preliminary background information. Since no sea turtle captures have been reported in traplines so far, their use may also reduce sea turtle bycatch, a possibility with potentially important implications for sea turtle conservation. However, in the previous studies on traplines, the number of turtles caught in the control gear (hooks) was extremely low [36,38], precluding robust conclusions about their effectiveness in reducing sea turtle bycatch.

The present study aims to complement those previous studies and to answer this specific question, i.e., whether traplines can also reduce sea turtle bycatch relative to conventional hooks while maintaining similar levels of commercial catch, and therefore whether traplines may represent a potential mitigation measure for drifting longline fisheries. Fishing is characterized by great variability, both in terms of environmental variables (e.g., season, temperature, area and oceanographic conditions) and in terms of technical and operational features (e.g., time of the day, soak duration, and gear configuration). To adequately account for such variability and understand how multiple factors can affect the performance of a fishing gear, data should be collected across a corresponding range of conditions. However, as a first step, the present study only focused on assessing whether traplines could reduce sea turtle bycatch compared with hooks, at least under specific circumstances, rather than evaluating their performance under all possible conditions, which would represent a second step. The specific hypotheses to be tested were: turtle catch rates are significantly lower with traplines than with hooks (H1) and commercial catches are not significantly lower with traplines than with hooks (H2). To this end, we evaluated the performance of traplines and traditional hooks in an area of the central Mediterranean characterized by high sea turtle catch rates, increasing the likelihood of having an adequate sample size for detecting possible differences.

## 2. Materials and Methods

### 2.1. Data Collection

**Figure 1:**
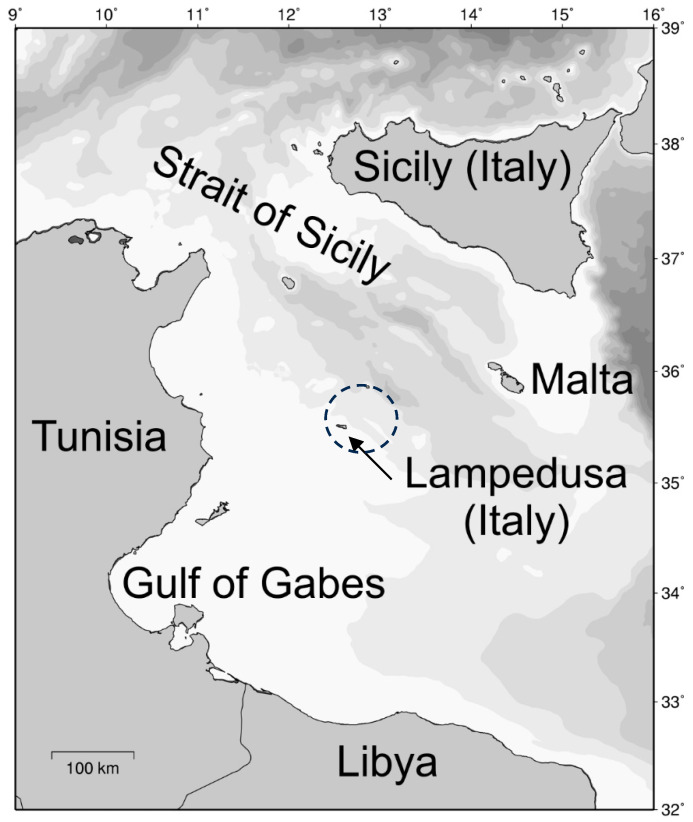
Study area. The approximative fishing area is shown by a dotted circle.

**Figure 2:**
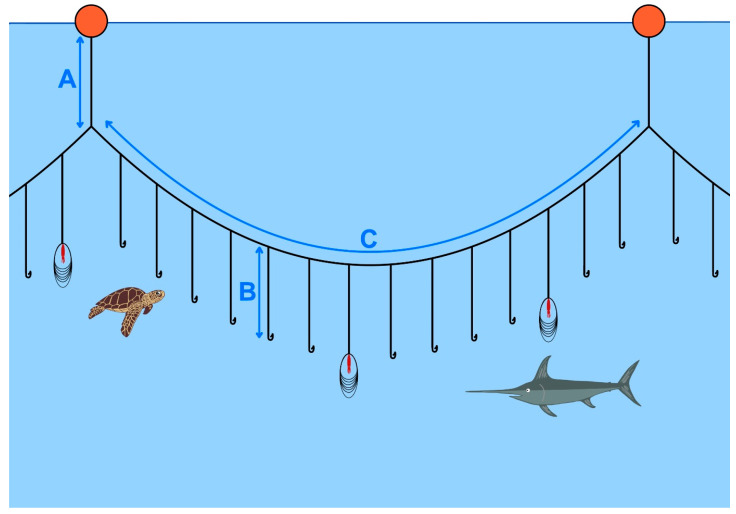
Longline configuration. A (length of the float line) and B (length of the branchline): 7 m; C (length of the line between floats): 350 m. On average, 12 hooks and 2 traplines are deployed between two floats, but their relative positions along the mainline were not consistent.

Under the approach outlined above, variability represented a potential confounding component, which we minimized by analysing 23 fishing sets conducted by a single fishing vessel, in a limited area (Figure 1), and during a relatively short period within the same year and season (2 June–13 August 2024). This vessel used a surface drifting longline mainly targeting swordfish. Within each fishing set, hooks and traplines were deployed in an alternating sequence along the mainline (on average, 12 hooks and 2 traplines are deployed between two floats; Figure 2), without a systematic pattern that would place either terminal gear deeper than the other on average, so that both terminal gears operated simultaneously under the same environmental and operational conditions. Fishing sets were conducted in the waters surrounding Lampedusa Island, Italy, where high turtle catch rates have been reported [10].

**Figure 3:**
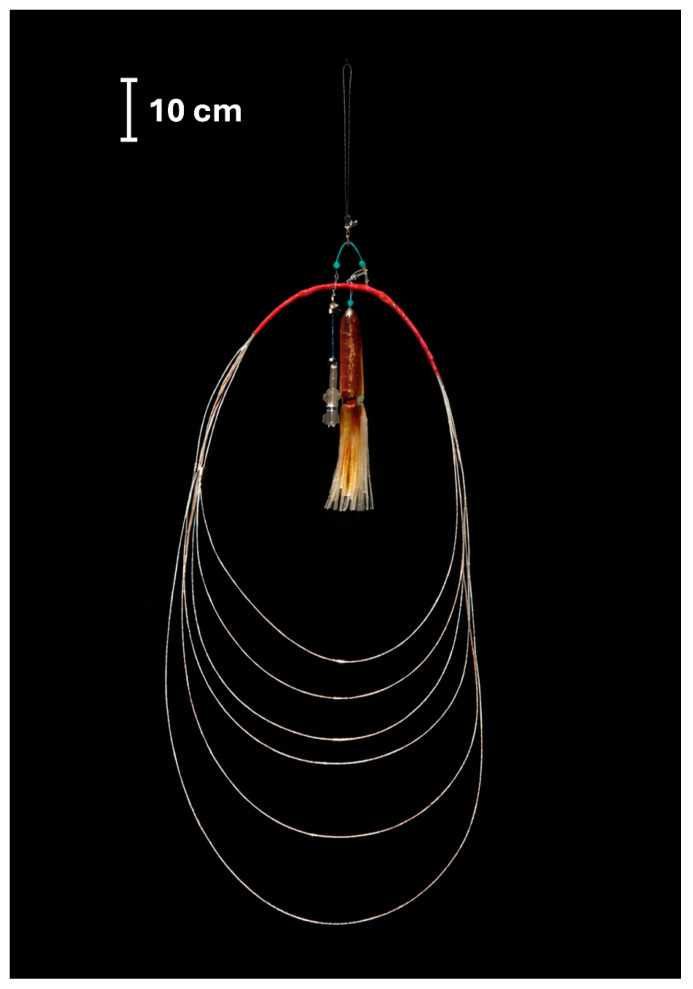
Photograph of a trapline of the type used in this study.

Each fishing set deployed 1700–1800 J hooks (size 4; Iuvella) and 250–300 traplines, for a total of 40,800 hooks and 6850 traplines deployed (Table 1) along a main line of approximately 50 km (see Figure 1 for the longline configuration). The approximate ratio between traplines and hooks was fixed in advance by the fisher. Each hook was baited with an artificial squid-like bait containing a piece of natural bait (*Sardina pilchardus* Walbaum, 1792). Each trapline consisted of six concentric nylon (1.1 mm) loops, with circumferences ranging from 140 to 215 cm, and only an artificial (soft plastic) squid-like bait (Figure 3) positioned at the centre, with a lead weight inside to keep it in place (Figure 3). A light (Banzai 1000-h, white flashing LED lamp; Banzai brand, distributed by Mareblu di Spina Grazia S.r.l., Acireale, Italy) was attached at the centre of each trapline.

For each fishing set and terminal gear type (hook and trapline), the following data were recorded and reported by the fisher: (i) the number of turtles caught (individuals), (ii) the commercial fish catch (kg), and (iii) the gear-specific fishing effort (number of hooks and traplines deployed).

Because data were reported by the fisher (on a voluntary basis) rather than by an independent onboard observer, results should be considered preliminary. The fisher had previously participated in similar bycatch research projects and was therefore considered reliable in recording and reporting catch data. Specifically, we assumed that the fisher had no reason to report fewer turtles and, more importantly for the aim of the study, no reason to report fewer turtles captured by one terminal gear than by the other. However, all captured turtles were landed and were examined by one of the authors (DF) to verify gear attribution, reducing the risk of misclassification for the primary conservation response variable (turtles). The presence of hooks or hook-related injuries confirmed capture by hooks, whereas the absence of a hook or hook-related injuries was consistent with capture by traplines, which do not leave visible signs.

### 2.2. Data Analysis

Turtle and fish catch per unit of effort (CPUE) were calculated using terminal gear units as the denominator, where terminal gear units were defined as individual hooks or traplines, each capable of capturing at most one animal at a time. This denominator was used as an operational measure of effort, because hooks and traplines represented the terminal capture devices deployed along the same mainline and any practical substitution between gears would occur at this level. We do not assume that one hook and one trapline are mechanically or biologically equivalent capture units; rather, CPUE per terminal gear unit was used to compare the relative operational performance of the two terminal devices deployed in this fishery configuration. For each set and gear type, turtle CPUE was calculated as the number of turtles captured divided by the number of terminal gear units deployed, whereas fish CPUE was calculated as the total mass of target fish captured divided by the number of terminal gear units deployed. Because hooks and traplines were deployed concurrently within each set, observations were treated as paired by set. The fishing set, rather than the individual hook or trapline, was therefore used as the unit of inference, because terminal gear units deployed within the same set shared the same fishing event and could not be considered independent replicates. Differences between gears were tested using paired randomisation tests based on sign-flipping of within-set differences. This framework was selected because it directly matched the paired design of the study: each fishing set provided an internal comparison between hooks and traplines under the same broad environmental and operational conditions. For each response variable, the paired difference *D* was calculated as:

$$D_{s}=X_{sP}-X_{sK}$$

where X is the response variable, s denotes the fishing set, and P and K denote traplines and hooks, respectively. Under the null hypothesis of no gear effect, gear labels are exchangeable within each set; equivalently, each $D_{s}$ can be multiplied by +1 or −1 with equal probability. Therefore, sign-flipping of the paired differences provides a direct test of whether the observed gear differences were larger than expected under random assignment of gear labels within sets. The median of the paired differences was used as the test statistic because the response variables were strongly non-normal, included many zeros in the case of turtle captures, and could be influenced by individual sets with unusually high catch values. For each test, we generated a permutation distribution by randomly flipping the signs of the observed $D_{s}$ across sets 10,000 times and recalculating the median of $D_{s}$ at each iteration. The observed median within-set difference was used as the test statistic. A two-sided *p*-value was calculated as the proportion of permuted medians with absolute values greater than or equal to the observed median. Direction was inferred from the sign of the observed median, with negative values indicating lower values for traplines than hooks and positive values indicating higher values for traplines. This permutation approach accounts for the paired design and avoids distributional assumptions, which was important because turtle captures were sparse and trapline catches were zero in nearly all sets.

As a complementary count-based sensitivity analysis for turtle captures, we also fitted a Poisson generalized linear model with the number of turtles captured as the response variable, terminal gear type as the explanatory variable, the logarithm of the number of terminal gear units deployed as an offset, while the fishing set was included as a fixed effect to preserve the paired comparison and control for set-specific variation. This model estimated the relative turtle capture rate of traplines compared with hooks while accounting for differences in gear-specific effort and the paired structure of the data.

To evaluate conservation efficiency relative to economic viability, we also tested whether traplines caught fewer turtles than hooks per unit of fish caught. Because turtles were recorded as counts whereas fish were measured as biomass (kg), we defined a scale-consistent metric: turtles per kg of fish. For each set s and gear type g, we computed the ratio *R*:

$$R_{sg}=\frac{T_{sg}}{F_{sg}}$$

where $T_{sg}$ is the number of turtles and $F_{sg}$ is the fish catch (kg) for set s and gear type g. This ratio quantifies turtle bycatch relative to fish catch. Although effort differed between gears and slightly among sets, effort was identical for turtles and fish within each gear and set, making the ratio internally consistent. Differences in this ratio between hooks and traplines were tested using the same paired randomisation procedure described above, with X=R. For all three response variables, effect size was reported as the observed median within-set difference between traplines and hooks. To quantify uncertainty around this effect size, 95% confidence intervals were calculated using paired bootstrap resampling, in which fishing sets were resampled with replacement and the median paired difference was recalculated for each bootstrap sample. The 2.5th and 97.5th percentiles of the bootstrap distribution were used as confidence limits. The three response variables were considered pre-specified and biologically distinct components of gear performance: turtle CPUE as the primary conservation response, fish CPUE as the commercial-performance response, and turtles per kilogram of fish as an integrated conservation-efficiency metric. Therefore, *p*-values are reported without formal multiple-comparison correction. As a sensitivity check, Holm-adjusted *p*-values were also calculated. All analyses were conducted in R (v. 4.5.1) [40].

## 3. Results

In total, 127 loggerhead sea turtles (*Caretta caretta*) and 9711 kg of fish were caught by hooks, whereas 1 turtle and 6580 kg of fish (almost exclusively swordfish according to the fisher) were caught by traplines (Table 1). The turtle caught by a trapline was entangled in a flipper and was in good condition.

Overall turtle catch rates were 3.11 turtles per 1000 hooks and 0.15 turtles per 1000 traplines. Turtle CPUE was significantly lower for traplines than for hooks, with a median within-set difference of −2.94 turtles per 1000 terminal gear units (traplines − hooks; 95% paired bootstrap CI: −3.53 to −2.78; paired permutation test, two-sided *p* < 0.001; n = 23 sets). The turtle capture rate of traplines estimated through the complementary count-based analysis was 0.047 times that for hooks (95% CI: 0.003–0.209; *p* < 0.01), corresponding to an estimated reduction of approximately 95% in turtle capture rate. Overall fish catch rates were 238 kg per 1000 hooks and 961 kg per 1000 traplines.

Fish CPUE was significantly higher for traplines than for hooks, with a median within-set difference of 688.89 kg per 1000 terminal gear units (traplines − hooks; 95% paired bootstrap CI: 396.86 to 972.22; paired permutation test, two-sided *p* < 0.01; n = 23 hauls). The turtle-per-fish ratio was significantly lower for traplines (0.00015 turtles kg^−1^ fish) than for hooks (0.013 turtles kg^−1^ fish), with a median within-set difference of −0.0132 turtles kg^−1^ fish (traplines − hooks; 95% paired bootstrap CI: −0.0152 to −0.0118; paired permutation test, two-sided *p* < 0.01). All three comparisons remained significant after Holm correction for multiple comparisons.

## 4. Discussion

The objective of this study was to evaluate whether traplines could reduce sea turtle bycatch relative to conventional hooks while maintaining commercial fish catch. The main finding was that traplines produced a very large reduction in turtle bycatch while still catching substantial fish biomass under the specific environmental and technical conditions evaluated. Turtle CPUE was approximately 95% lower for traplines than for hooks, whereas fish CPUE was higher for traplines. This study provides the first preliminary empirical evidence of the potential value of traplines as a mitigation technology for sea turtle bycatch in pelagic longlines targeting swordfish.

### 4.1. Implications for Fisheries Management and Conservation Policy

In the specific environmental and technical context of this study, traplines resulted in an almost complete elimination of turtle bycatch, with only a single turtle captured across all fishing sets, in contrast to hooks. This is consistent with previous studies in which no turtles were captured by traplines, although the low number of turtles captured by hooks in those studies did not allow for strong inference [36,38].

Given these outcomes, it is difficult to identify alternative gear modifications capable of achieving a greater reduction in turtle bycatch under similar fishing conditions. Deep-setting longlines represent the only partially comparable mitigation approach, as hooks are deployed at depths generally less accessible to turtles [19], although some deep-diving species can also be captured [13]. However, turtles remain vulnerable during gear deployment and retrieval, when hooks pass through shallower waters. Furthermore, enforcement of deep-setting requirements can be challenging, because the gear characteristics determining fishing depth are not always straightforward to verify during port inspections. In contrast, traplines differ structurally from hooks and this may facilitate identification at port and general compliance monitoring.

Traplines produced 40% of the fish biomass despite representing only 14% of terminal gear units, suggesting higher per-unit catch performance than hooks, consistent with patterns reported in recent preliminary technical reports [36,37,38]. When directly compared with hooks, traplines yielded a markedly lower number of turtles per unit of fish caught, highlighting their conservation potential. If such performance is confirmed in a variety of fishing contexts, the number of traplines in a longline could be adjusted to provide a commercial yield similar to that of a traditional longline with hooks but with much lower levels of turtle bycatch. Even if baited hooks indirectly increased trapline catches, a practical solution would be to retain baits while eliminating hooks (i.e., deploying branchlines with bait only or adding organic bait to the trapline). In either case, removing hooks—whether bait is retained or not—would effectively eliminate turtle hooking while allowing fishers to potentially maintain commercially viable levels of catch. This consideration is important from a conservation and management perspective because practical gear modifications may involve a combination of structural and operational changes, and the primary question is whether the tested configuration can reduce turtle bycatch while maintaining commercial catch volumes.

### 4.2. Limitations of the Study

The observed contrast between hooks and traplines strongly supports a lower turtle capture rate for traplines under the conditions studied. However, because only one turtle was captured by traplines, the exact magnitude of the trapline turtle capture rate is uncertain, and additional data are needed to determine how consistently this reduction occurs across other fishing contexts.

Because hooks and traplines were deployed at unequal effort, raw catch totals should be interpreted descriptively; the comparison between gears is based on effort-standardized CPUE and paired within-set differences, rather than on raw totals alone. Moreover, because commercial catch was reported as total biomass by the fisher and was not independently verified or separated by species for each terminal gear, these results should be interpreted as preliminary evidence of higher biomass catch per terminal gear unit rather than as a direct estimate of gear-specific commercial value.

Because hooks and traplines were deployed simultaneously along the same mainline, the present study estimates trapline performance only within this mixed-gear configuration and not in isolation. The mixed deployment of hooks and traplines therefore did not allow for the estimation of trapline catch rates of commercial species in the absence of hooks. It is therefore possible that baited hooks attracted fish in proximity to the longline, indirectly increasing trapline catches. Consequently, the higher fish CPUE observed for traplines should not be interpreted as evidence that trapline-only longlines would necessarily produce the same catch rates.

Moreover, the two terminal gear treatments differed in more than capture mechanism alone: hooks were deployed with artificial squid-like bait plus natural sardine bait, whereas traplines were deployed with artificial squid-like bait and LED lights. Previous studies indicate that the use of light is optional and may be included or not at the fisher’s discretion [33,37,38]. Therefore, the present study cannot isolate the independent effects of hook absence, bait type, or light use. Instead, the results should be interpreted as an evaluation of the trapline terminal-gear configuration as operationally deployed in this fishery, rather than as a test of any single gear component.

The paired design controlled for set-level environmental and operational variation because hooks and traplines were deployed simultaneously along the same mainline. However, variables such as sea surface temperature, current conditions, effective fishing depth, and soak or haul duration were not measured or analysed explicitly. Similarly, this study is based on a single vessel and generalization to the wider fleet requires replication across multiple vessels to account for variability associated with skipper decisions, crew handling practices, vessel configuration, or other fishing practices. Naturally, it cannot be excluded that the present results are due to a fortunate unique combination of environmental and technical variables and may not occur in different contexts.

### 4.3. Implications for Future Research on Hookless Fishing Gear

A valuable next step would also be to conduct dedicated trials using traplines alone to quantify species-specific fish catch rates and associated commercial value in the absence of surrounding baited hooks and to determine a commercially viable configuration, for example, by adjusting the number of traplines per unit of longline length and assessing whether additional baiting is required. Specific trials are needed to assess whether the magnitude of the trapline effect varies across environmental or operational contexts, such as sea surface temperature, current conditions, effective fishing depth, and soak or haul duration. Trials conducted in fisheries with different technical features and in areas with different environmental features are needed to confirm the present findings in wider fishery contexts.

Future studies should record species composition, individual biomass, and market value separately for hooks and traplines. If the objective is to identify which component of the trapline configuration drives the observed differences, controlled trials would be required to separate the effects of capture mechanism, bait type, and light use.

If the efficiency of traplines for catching swordfish is confirmed, their potential impact on the target species should also be evaluated. Moreover, preliminary studies conducted to date have only focused on longlines targeting swordfish and blue shark [36,37,38]. Consequently, the performance of traplines should also be evaluated in fisheries targeting other commercial species (e.g., albacore tuna), where catch dynamics, species behaviour, and operational practices may differ. Finally, in this study, bycatch data were only available for sea turtles. Therefore, from a multispecies and ecosystem-based conservation perspective, the potential effects of traplines on other species of conservation concern should also be investigated.

## 5. Conclusions

The present results provide strong, although preliminary, indications of the conservation potential of traplines as a technically feasible measure capable of dramatically reducing—and potentially eliminating—the impact of swordfish drifting longlines on sea turtles. Both working hypotheses were supported by the results: turtle catch rate, but not commercial catch rate, was lower with traplines than with hooks. Moreover, traplines lack hooks; consequently, turtles can only become entangled rather than hooked. This may facilitate rapid release by fishers and reduce the risk of post-release mortality.

## Figures and Tables

**Table 1:** Number of sea turtles (n) and biomass of commercial fish (kg) captured during 23 pelagic longline fishing sets in waters around Lampedusa Island.

Fishing Set (2024)	N Hooks	N Traplines	N Turtles Caught by Hooks	N Turtles Caught by Traplines	Kg Fish Caught by Hooks	Kg Fish Caught by Traplines
2 June	1700	250	3	0	110	80
8 June	1700	300	5	0	70	60
21 June	1700	300	4	0	270	140
27 June	1700	300	7	0	490	315
28 June	1700	300	6	0	359	240
29 June	1700	300	6	0	487	205
1 July	1800	300	7	0	590	390
6 July	1800	300	5	0	490	350
7 July	1800	300	4	0	880	420
10 July	1800	300	6	0	395	690
12 July	1800	300	3	0	430	380
15 July	1800	300	3	0	390	500
18 July	1800	300	8	1	780	510
19 July	1800	300	5	0	380	220
21 July	1800	300	8	0	340	310
26 July	1800	300	6	0	440	280
27 July	1800	300	5	0	510	230
28 July	1800	300	4	0	500	390
1 August	1800	300	8	0	390	410
7 August	1800	300	6	0	510	160
8 August	1800	300	5	0	380	100
11 August	1800	300	7	0	220	90
13 August	1800	300	6	0	300	110
Total	40,800	6850	127	1	9711	6580

## Data Availability

The data presented in this study are available in Table 1 of this article.
